# A Noble Profession: Nursing the Sick

**Published:** 1886-10-23

**Authors:** 


### A Noble Profession: Nursing the Sick.

**Half a Century Ago.**

Since Mrs. Fry, the eminent Quakeress and philanthropist, established, nearly half a century ago, the Association of Nursing Sisters, there has been a rapid, nay a marvellous, development in the art of nursing. Nursing is happily now no longer regarded as a calling fit only for women of the lower orders of society. In most of our great hospitals and associations for promoting trained nursing, ladies of gentle birth may be found who have dedicated the most useful period of their lives to the alleviation of the suffering. Elizabeth Fry has been justly styled "the female Howard." Though many of her sex have since nobly followed in the path she opened out, we must not forget the fact that she was the pioneer. Grand indeed were her exertions, and undaunted was her zeal in ministering to the lot of the wretched. She saw before her a world of suffering, and, undeterred by the immensity of her self-imposed task, she determined to devote her purse and her life to the noble cause.

**Later Developments.**

But the labours of this true heroine, though they bore good and lasting fruit, were but as a drop in the ocean. After she had passed away another heroine arose, whose grand exertions have played no small part in placing the art of nursing in the position it occupies to-day. The opportunity makes the man or woman. Great events call forth great efforts on the part of the best of our race. It was so with nursing. The terrible narratives which reached England of the sufferings of our soldiers in the Crimea made many hearts bleed. Florence Nightingale came forward, with some self-sacrificing ladies. The work of these ministering angels is now a matter of history. To these pioneers belongs the lion's share of the credit which attaches to the systems of skilled nursing to be now found in most civilised countries throughout the world. The school which Miss Nightingale founded, and the scientific principles which she laid down, completely revolutionised the art of nursing. At home, Mrs. Wardroper, at St. Thomas's Hospital, and Miss Jones, at Liverpool, established a system of hospital nursing which has ramified throughout the English-speaking world. Those who initiated this great humanising work deserve well of their day and generation. This is but a truism, but its enforcement is demanded at the present time by the apparent neglect to grasp the facts of the situation which seems to prevail everywhere.

**Nurses and their Training.**

The nurse is the doctor's handmaiden. Upon her skill, judgment, and attention depends to a very large extent the patient's convalescence. Nursing, like any other art, can be learnt only by dint of hard and constant practice. But there are many women whom no amount of assiduity to duty would make good nurses. A nurse's nature must be sympathetic; she must possess tact and intelligence; she must have a knowledge of human nature; but, above all, she must have a real love for her profession. We can keep ledgers efficiently, but yet hate book-keeping; we can be proficient with the Morse printer and the single needle, and yet entertain no liking for the calling of the telegraphist. It is far otherwise with nursing. But, granted a love for her calling, and the qualifications which we have named, a course of careful training is absolutely necessary. The London and some Provincial hospitals afford excellent and ample facilities for the attainment of a thorough practical knowledge of every detail of the art. This fact is not appreciated as it ought to be by those who need and employ nurses. Were such the case, the nursing of the sick in private houses would be much better done than it is at present. Many hospitals are trying to bring this fact home to the public conscience, and we shall do our best to enforce the lesson.

